# Wavelet geographically weighted regression for spectroscopic modelling of soil properties

**DOI:** 10.1038/s41598-021-96772-z

**Published:** 2021-09-01

**Authors:** Yongze Song, Zefang Shen, Peng Wu, R. A. Viscarra Rossel

**Affiliations:** 1 School of Design and the Built Environment, Curtin University, GPO Box U1987, Perth, WA 6845 Australia; 2 Soil and Landscape Science, School of Molecular and Life Sciences, Curtin University, GPO Box U1987, Perth, WA 6845 Australia

**Keywords:** Ecology, Environmental sciences

## Abstract

Soil properties, such as organic carbon, pH and clay content, are critical indicators of ecosystem function. Visible–near infrared (vis–NIR) reflectance spectroscopy has been widely used to cost-efficiently estimate such soil properties. Multivariate modelling, such as partial least squares regression (PLSR), and machine learning are the most common methods for modelling soil properties with spectra. Often, such models do not account for the multiresolution information presented in the vis–NIR signal, or the spatial variation in the data. To address these potential shortcomings, we used wavelets to decompose the vis–NIR spectra of 226 soils from agricultural and forested regions in south-western Western Australia and developed a wavelet geographically weighted regression (WGWR) for estimating soil organic carbon content, clay content and pH. To evaluate the WGWR models, we compared them to linear models derived with multiresolution data from a wavelet decomposition (WLR) and PLSR without multiresolution information. Overall, validation of the WGWR models produced more accurate estimates of the soil properties than WLR and PLSR. Around 3.5–49.1% of the improvement in the estimates was due to the multiresolution analysis and 1.0–5.2% due to the integration of spatial information in the modelling. The WGWR improves the modelling of soil properties with spectra.

## Introduction

Soil properties are critical indicators of ecosystem function^1,2^. They can directly indicate the quality of ecosystem services, including food and energy production, plant growth, carbon storage, regulation of greenhouse gas emissions and climate change^3–7^. Soil organic carbon, clay content and pH are essential soil properties affecting soil nutrient supply and plant development^8^. However, the measurement of these soil properties remains challenging because conventional analytical methods are time-consuming and expensive^9,10^. Diffuse reflectance soil spectroscopy, for example, using visible and near infrared (vis–NIR) spectra, has been proposed as a means to overcome those issues. The physcial basis of vis–NIR spectroscopy relies on overtones and combination bands from fundamental molecular vibrations of bonds in molecules of soil consituents, which occur in the mid infrared region^11,12^. Increasingly, the method has been used to estimate soil properties and to estimate their values more rapidly and cost-efficiently than conventional laboratory analytical methods^13,14^. Another advantage of the method is that a vis–NIR spectrum can be used to simultaneously characterise multiple soil properties^4^.

Methods for modelling continuous soil properties with highly collinear spectra include multivariate statistics and machine learning. The most common statistical methods are principal component regression (PCR)^15,16^ and partial least squares regression (PLSR)^17,18^. Different machine learning algorithms also have been used, including support vector machines, artificial neural networks, random forests and other regression trees^19^. More recently, convolutional neural network (CNN) and other deep learning architectures are also being developed^20–22^.

Wavelets have been successfully used with spectra in soil science and other fields of research^4,23,24^. Studies have demonstrated that the discrete wavelet transform (DWT) can improve the analysis of soil diffuse reflectance spectra for the prediction of soil properties^14^. They showed that multiresolution analysis (MRA) of soil diffuse reflectance spectra could identify different spectral features that occurred over different resolutions (or scales). They also showed that the highly collinear spectra could be transformed into a smaller number of orthogonal wavelet coefficients that produced more parsimonious and accurate multivariate calibrations.

Soil properties, like other natural phenomena, vary spatially and at different scales^25–27^. This variability is due to complex interactions between the environmental factors that affect the formation and distribution of soil^28^. The incorporation of spatial information in aspatial models can improve the accuracy of their predictions^29^. Spectroscopic modelling of soil properties often ignores geography and the spatial dependence of soil properties. Only a few studies have tried to account for geography in spectroscopic modelling. For example, the states or territories of Australia were used as categorical variables to account for any variance in the modelling of Australian soil spectra resulting from geography^30^. Sila et al.^31^ used regression–kriging to predict soil properties with mid-infrared spectra of soil samples, where residuals from a regression fit were informed using variograms.

Geographically weighted regression (GWR) might be a useful tool for modelling spectra and accounting for the geographic relationships and spatial non-stationarity in the data^32^. GWR, developed by^33^, supports locally varied regression parameter estimates for each explanatory variables across space^34,35^. The recent advances in the methodology and applications of GWR have helped to acquire new understanding of spatial processes. For example, basic GWR has been adapted for improved local inference of soil property data^36^, it has been adapted to a multiscale form^37,38^, to address issues of local multicollinearity^39^, and to down-weight the influence of outliers for robustly estimating the variability of local coefficients in social data^40,41^. These studies demonstrate the power and versatility of GWR for measuring spatial non-stationarity^37,38,42,43^. As such, GWR has been used in various fields of research, including ecology and environment^44^, climate^45^, social science^46^ and public health^47^.

Here, we propose to combine wavelets with geographically weighted regression (WGWR). Our hypothesis is that the spectral modelling of soil properties can be improved by accounting for the multiresolution information in the spectra and the spatial variations of the data. Thus, our aim is to demonstrate the implementation of WGWR for modelling soil properties with vis–NIR spectra and to evaluate the performance of the WGWR by comparing its predictions to those from PLSR and WLR. Experiments were conducted using a spectral library from the south-western West of Australia (WA).

## Results

**Figure 1:**
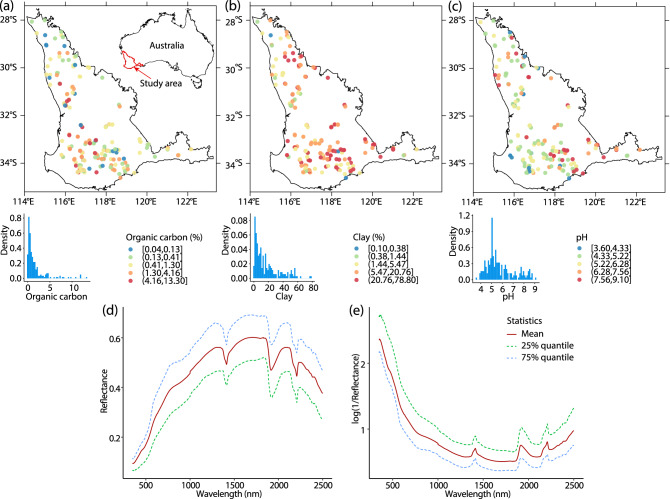
Spatial and density distributions of soil organic carbon (**a**), clay (**b**) and pH values (**c**) in the study area, and statistics of vis–NIR spectra: (**d**) Reflectance; (**e**) log(1/Reflectance).

The spatial distributions of the organic carbon, clay content and pH data at the sampling locations are shown in Fig. 1a–c. The statistical summary of soil properties is shown in Table 1. In the study area, the mean soil organic carbon is 1.61%, mean clay content is 16.64% and mean pH is 5.77. Maps of spatial distributions, density figures, and statistical summaries of soil properties indicate their spatial variation across the study area.

**Table 1:** Descriptive statistical summary of the soil properties. SD is the standard deviation, CV the coefficient of variation and Skew. is the skewness coefficient.

Soil property	No.	Mean	SD	Min.	Median	Max.	CV (%)	Skew.
Organic carbon (%)	222	1.61	2.33	0.04	0.77	13.30	1.45	2.83
Clay (%)	220	16.64	16.93	0.50	10.10	78.80	1.02	1.34
pH	223	5.77	1.27	3.60	5.40	9.10	0.22	0.86

Figure 1d, e shows the measured and transformed spectra. The broad absorptions between 350–1100 nm are associated with the iron oxides hematite or goethite, but also with organic carbon^48^. The wavelengths near 1412 nm are generally associated with the first overtone of hydroxyl stretching modes of water and minerals^49^. The wavelengths near 1917 nm are linked with hydroxyl and H-bonding hydroxyl stretching vibrations of water molecules and mineral constituents of water^50^. The neighbouring wavelengths near 2207 nm are related to clay minerals^51,52^.

### Wavelet Geographically Weighted Regression (WGWR) of soil properties

#### Multiresolution analysis

**Figure 2:**
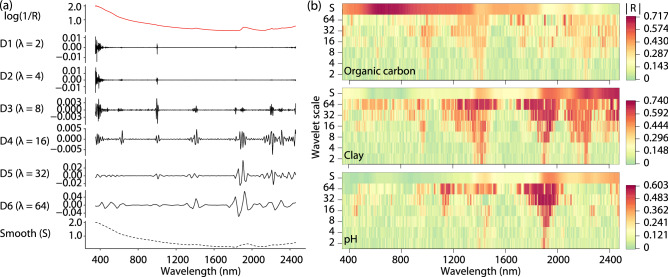
Multiresolution analysis of vis–NIR spectra: Smooth component (S) and details (Di, i = 1, 2, 3, 4, 5, 6) at different wavelet scales (**a**), and absolute values of correlation coefficients between soil properties (organic carbon, clay and pH) and wavelets of vis–NIR spectra (**b**).

The MRA of a vis–NIR spectrum shows six scales with detailed coefficients and a smooth component at the coarsest scale (Fig. 2a). The details at the different wavelet scales reveal the multiresolution features of soil spectra. At the finest scales $\lambda$ = 2 and 4, the high frequency elements of the spectra occur at the interface between the three detectors in the spectroscopic sensor, where the signal is ‘noisier’. At the medium scales $\lambda$ = 8 and 16, the wavelet coefficients depict the edges of the absorptions of the soil constituents near 595 nm, 1007 nm, 1415 nm, 1831 nm, 1903 nm, and 2207 nm. At the coarse scales $\lambda$ = 32 and 64, the wavelet coefficients represent the broader absorptions of soil consituents primarily near 1400 nm and 1900 nm. The MRA results indicate that wavelet transformation can effectively identify the multiresolution local features of soil spectra.

#### Optimal identification of wavelets

Figure 2b shows the absolute correlations (|*R*|) between soil organic carbon, clay and pH, and the wavelet coefficients at the different wavelet scales. The larger |*R*| values occur at different wavelengths and wavelet scales, showing the multiresolution features in the spectra. For organic carbon, the largest |*R*| values at the smooth and detail components occur near 632 nm, 1894 nm, 1984 nm, 1953 nm, 985 nm, 1004 nm and 1003 nm. For clay content the largest |*R*| values occur near 2246 nm, 2399 nm, 2455 nm, 1927 nm, 1893 nm, 1892 nm and 1890 nm, and for pH near 1940 nm, 1949 nm, 1862 nm, 1906 nm, 1905 nm, 1892 nm and 1890 nm. The wavelets around these wavelengths show greater correlations with the soil properties as they represent absorptions due to the mineral and organic composition of soil^53^.

**Figure 3:**
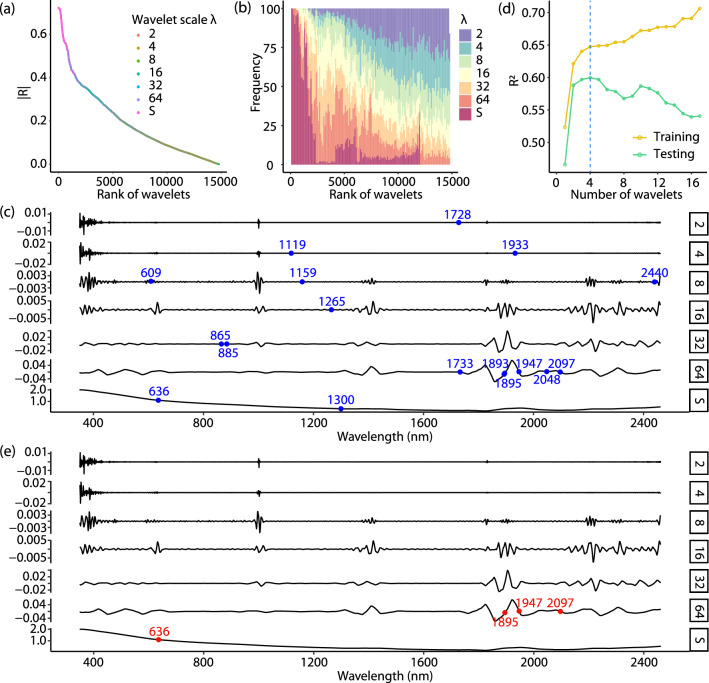
Process of selecting optimal combinations of wavelets for soil organic carbon prediction: (**a**) Ranked wavelets by the absolute values of correlation coefficients; (**b**) Statistical summary of wavelet scales by the rank of wavelets; (**c**) Wavelets selected by a multicollinearity analysis where the maximum VIF is lower than 10; (**d**) Ten-fold cross validation for selecting wavelets with the maximum testing R$^2$; and (**e**) Selected optimal combinations of wavelets for explaining soil organic carbon.

Figure 3 illustrates the procedure for selecting the optimal combinations of wavelet coefficients for soil organic carbon. The selection of optimal coefficients for soil clay and pH followed a similar processes. According to the distributions of |*R*| values, wavelets are ranked from the highest to the lowest |*R*| values (Fig. 3a). Figure3b shows the frequency of the ranked wavelets grouped by wavelet scales. The statistical summaries indicate that the wavelets at coarse scales tend to be more correlated with soil organic carbon compared with the wavelets at fine scales.

**Figure 4:**
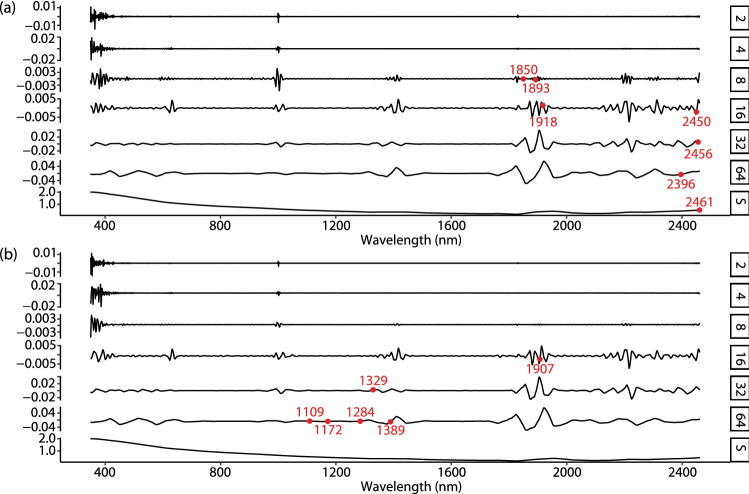
Optimal combinations of wavelets for explaining soil clay (**a**) and pH values (**b**).

Figure 3 c shows the wavelets selected by the multicollinearity analysis with the threshold that the maximum variance inflation factor (VIF) value is lower than 10 (see "Methods" section). In this step, 17 wavelet coefficients were selected. This shows that vis–NIR spectra are highly collinear and redundant. The number of explanatory wavelets are much reduced and the multicollinearity is eliminated. Figure 3 d shows the result of the fitness of the wavelet coefficient selection, which we performed by a ten-fold cross validation. With increases in the number of wavelet coefficients, the fitness of training data gradually increased, but that of testing data increased initially and decreased after four coefficients. This shows that the combination of the four coefficients was optimal for modelling soil organic carbon (Fig. 3e). The selected optimal combinations of wavelet coefficients for clay and pH are shown in Fig. 4.

#### Geographically weighted regression

**Figure 5:**
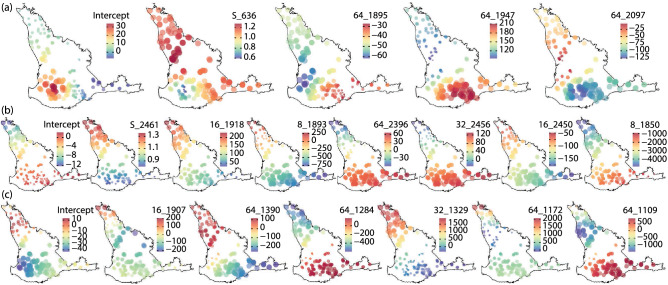
Distributions of local coefficients of wavelets in WGWR of soil organic carbon (**a**), clay (**b**), and pH values (**c**). Sizes of points indicate absolute values of coefficients.

Due to the spatial non-stationarity of soil properties, GWR is used to model the relationships between soil properties and the selected optimal combinations of wavelets. Figure 5 shows spatial distributions of local coefficients of wavelets in WGWR models, where significance of local coefficients were tested but not shown on the maps. The coefficients of both training and testing data are combined on maps of Fig. 5. The maps of local coefficients indicate spatially variable coefficients of wavelets across the study area for predicting soil organic carbon, clay and pH values. The spatially variable local coefficients also reveal the spatial non-stationarity of the relationships between soil properties and spectra data.

### Comparing WGWR to other methods

**Figure 6:**
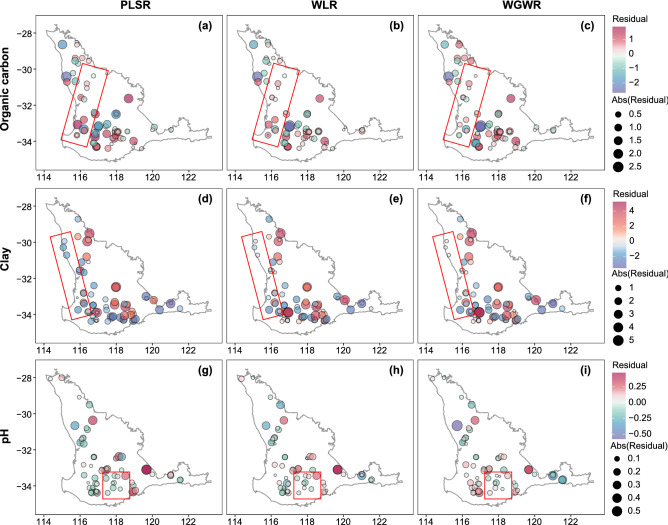
Maps of residuals in PLSR, WLR, and WGWR for the test data of soil organic carbon (**a**–**c**), clay (**d**–**f**), and pH values (**g**–**i**). Regions marked with a red rectangular outline demonstrate the difference of the models and the accuracy of the estimates.

Figure 6 shows maps of the PLSR, WLR, and WGWR residuals calculated on the test dataset for soil organic carbon, clay and pH, respectively. The maps indicate that the absolute values of the residuals are smaller for WLR and WGWR, respectively, compared with PLSR, due to the wavelet-based multi-resolution analysis. In addition, compared to the WLR, the residuals from the WGWR are smaller.

The validation results of the PLSR, WLR and WGWR are given in Table 2. In the PLSR, the (R$^2$) of the models derived using the training data of soil organic carbon, clay and pH are 0.547, 0.674 and 0.445, and the R$^2$ of the models when generalised on the test data are 0.477, 0.389 and 0.347, respectively. Due to the incorporation of multi-solution information, the WLR models performed better than PLSR. Compared to PLSR, the R$^2$ of the WLR of organic carbon, clay and pH increased by 22.4%, 49.1% and 3.5%, and the RMSE reduced by 10.8%, 17.1% and 0.9%, respectively. The incorporation of geographical information helped to improve the accuracy of the spectroscopic soil property estimates. In the WGWR, the training R$^2$ of soil organic carbon, clay and pH are 0.702, 0.678 and 0.414, and the test set R$^2$ values are 0.590, 0.587 and 0.378, respectively. Compared to WLR, the R$^2$ of the WGWR estimates of organic carbon, clay and pH increased by 1.0%, 1.2% and 5.2%, and the RMSE decreased by 0.7%, 0.8% and 1.5%, respectively. Thus, compared to PLSR, the R$^2$ of the WGWR estimates of organic carbon, clay and pH increased by 23.6%, 50.9% and 8.8%, and their RMSE decreased by 11.4%, 17.8% and 2.4%, respectively.

**Table 2:** Assessment statistics for the validation of PLSR, WLR and WGWR.

Soil property	Statistic	PLSR		WLR		WGWR	
		Training	Testing	Training	Testing	Training	Testing
Organic carbon	R$^2$	0.547	0.477	0.646	0.584	0.702	0.590
	AICc	378.4		348.7		332.2	
	MAE	0.630	0.744	0.527	0.613	0.473	0.626
	RMSE	0.793	0.947	0.701	0.845	0.643	0.839
Clay	R$^2$	0.674	0.389	0.655	0.581	0.678	0.587
	AICc	351.1		343.2		341.7	
	MAE	0.506	0.746	0.523	0.641	0.505	0.633
	RMSE	0.674	1.059	0.693	0.878	0.670	0.871
pH	R$^2$	0.445	0.347	0.408	0.359	0.414	0.378
	AICc	− 83.3		− 90.6		− 114.1	
	MAE	0.731	0.621	0.761	0.618	0.757	0.614
	RMSE	1.004	0.862	1.038	0.854	1.032	0.841

In addition, the Akaike information criterion (AICc) also demonstrate the improved accuracy and parsimony of WGWR.

## Discussion

This study proposes a WGWR to more accurately estimate soil properties using reflectance spectra. We demonstrate that the integration of an MRA of reflectance spectra and spatial non-stationarity in the relationships between soil properties and spectra can improve the spectroscopic modelling of soil properties. The advantages of WGWR are the improved prediction accuracy, fewer spectral variables with reduced multicollinearity, and more robust estimates compared to PLSR and WLR.

The assessments of the soil organic carbon, clay and pH estimates indicate that the multiresolution features of spectra modelled by wavelet-based MRA can improve the skill of the modelling by 3.5–49.1%. Viscarra Rossel & Lark^14^ developed the modelling of soil properties with wavelets using an MRA. Compared to the approach by Viscarra Rossel & Lark, this study provides an alternative framework for the selection of coefficients. Here, we use correlation rather than variance for the ranking of coefficients and the VIF for eliminating multicollinearity, followed by ten-fold cross validation to minimise overfitting. As a result, the number of predictors were much reduced for modelling with WLR and WGWR. Two-thousand-one-hundred-and-fifty-one vis–NIR wavelengths were used in the PLSR, but only 4, 7 and 6 wavelets were selected for modelling soil organic carbon, clay and pH with WLR and WGWR.

The consideration of spatial non-stationarity in the WGWR, reduced errors and improved the accuracy of the models. Thus we show that WGWR can improve the modelling of soil properties with spectra by accounting for both multi-resolution information and spatial non-stationarity.

Our results suggest that, if spatial information is available, geographical characteristics of soil properties should be considered and used in spectroscopic modelling of soil properties.

Reflectance spectroscopy is an efficient and cost-efficient approach for rapidly estimating soil properties. We developed the WGWR that effectively integrates the multiresolution characteristics of soil vis–NIR spectra, the process of optimal wavelets identification and the spatial variations of soil properties and the spectra. Compared to PLSR and WLR, WGWR produced more accurate estimates of soil organic carbon, clay content and pH. The models were more parsimonious and thus the danger of multicollinearity of spectral variables and overfitting was eliminated. Improved modelling of soil properties with spectra, like we have done here, can also provide insights of geographical characteristics in soil-related ecosystems services, climate responses and sustainable development. Future studies might investigate the use of other geospatial methods for use with soil spectra, such as as kriging with external drift^54^.

## Methods

### Study area and soil observations

The study region in the south west of WA covers about 252,100 km$^2$. It represents diverse land uses, including cropping, native forests and nature conservation. It is one of the primary agricultural production regions in Australia^55^. In 2018–2019, the gross value of agricultural production (GVAP) in WA, primarily in the South West Agricultural Area, was about 18% of the national GVAP^56^. The primary grains produced in this area include wheat, barley, canola, lupins, oats, and field peas, where wheat account for 65% of annual grains in WA^57^. Understanding the soil properties is essential for agricultural and environmental management.

We used a set of 226 soil samples collected within the study area. The shortest distances between soil samples and their nearest neighbours vary from 0.24 to 84.16 km. Among the samples, 20.4% of the sample points have neighbour samples within 1 km, and 33.2% of the sample points are the only samples within a radius of 10 km. At each sampling location, samples were taken at multiple depths from the surface down to 135 cm. The soil properties measured included soil organic carbon, clay content and pH measured in water. The analytical methods used to measure these soil properties are described in Rayment^58–60^. To derive a more consistent dataset for the modelling (described below), at the each sampling location, we took a weighted average of the soil properties from different depths, using the depths as weights.

The statistical distributions of soil organic carbon and clay content were negatively skewed so they were transformed to approximate normality using logs. Outliers were identified by setting thresholds of 2.5 standard deviations from the mean values^61^. Values that exceeded the threshold were removed. As a result, 4, 6 and 3 outliers were removed from the organic carbon, clay and pH data, respectively.

### vis–NIR spectroscopy

The vis–NIR reflectance spectra of 226 soil samples were measured with a Labspec vis–NIR spectrometer (PANalytical Company, Boulder, CO., USA). The spectral range of the spectrometer spans from 350 to 2500 nm, and it has a spectral resolution of 3 nm at 700 nm and 10 nm at 1400 and 2100 nm. Measurements were made with a high-intensity contact probe (also from PaNalytic) that uses a halogen bulb ($2901\pm 10$ K) for illumination. The contact probe measures a spot of diameter 10 mm, and it is designed to minimize errors associated with stray light. The sensor was calibrated with a Spectralon® white reference panel once every ten measurements. For each soil sample, 30 spectra were averaged to minimize noise and so to maximize the signal-to-noise ratio. The measurements were made following the protocols described in Rossel^4^. Spectra were recorded with a sampling resolution of 1 nm so that each spectrum comprised reflectances at 2151 wavelengths. The measured reflectances, R, were first converted to apparent absorbance as log10(1/R).

### Wavelet geographically weighted regression

**Figure 7:**
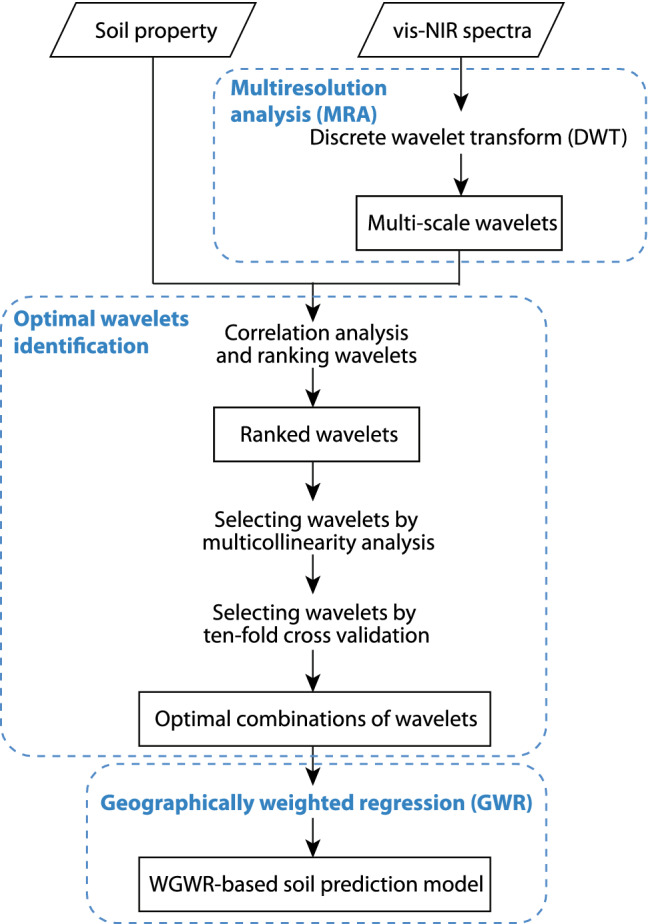
Flowchart of the wavelet geographically weighted regression (WGWR) model for soil prediction.

To improve the modelling of soil properties with spectra, we developed a WGWR model. It integrates the multiresolution information in the spectra and the spatial variations of soil properties. The WGWR model consists of three steps: (1) decomposition of the vis–NIR spectra with a DWT and MRA, (2) selection of an optimal set of wavelet coefficients for the regression, and (3) GWR. The workflow is shown in Fig. 7.

First, we decomposed each vis–NIR spectrum using the DWT and MRA to reveal the multiresolution nature of the spectra. For the decomposition, we used the Daubechies wavelet with 4 vanishing moments. The MRA is implemented via a pyramid algorithm^62^, in which a spectrum is decomposed into the detail components ($D_i$) at different wavelet scales ($\lambda _i$) up to a coarsest scale, when a smooth or approximation component (*S*) is obtained. In this study, the spectrum beyond its boundaries, including the start and end of the data, is assumed to be a symmetric reflection of the spectrum^63^. The sum of the detail and smooth components is the original spectrum. Viscarra Rossel and Lark^14^ provide a description of the approach for the analysis of soil spectra. The decomposition was performed, as above, for the vis–NIR spectra of all samples.

Second, to identify the optimal wavelet coefficients for modelling, we correlated the soil properties to the wavelet coefficients and recorded the Pearson correlation coefficient. We then ranked the wavelet coefficients according to the absolute values of correlation coefficients (|*R*|). A multicollinearity analysis was then performed using a VIF, a measure of multicollinearity of variables in a regression model, to discard wavelet coefficients that were highly correlated. Highly correlated explanatory variables can lead to unstable coefficients and a less accurate regression^64,65^. From the ranked set of coefficients, the wavelet with the largest |*R*| was selected as the first explanatory variable to use in the regression to estimate the multicollinearity among wavelets. Then, wavelet coefficients from the ranked list were sequentially added to the first, and a linear regression performed. If the VIF was smaller than 10, that wavelet coefficient is selected, but if it was larger than 10, then that coefficient was removed. The procedure continued sequentially and the final selected coefficients are uncorrelated and with a VIF smaller than 10. The remaining selected wavelet coefficients, were sequentially added one at a time to perform regressions using a ten-fold cross validation. We did this to eliminate overfitting in the assessments and modelling. The average cross validation R$^2$, and the number of wavelet coefficients were compared to derive the optimal number of coefficients with the highest average cross validation R$^2$. Thus, the final selected wavelet coefficients were the optimal combination for each of the modelled soil properties.

Third, a GWR is applied to characterise geographically local relationships between soil property and the optimal combination of wavelets derived from reflectance spectra. Soil properties are spatially correlated^66,67^. The GWR models enable locally varied estimates of coefficients for all explanatory variables in the regression. The spatial non-stationarity of soil properties is examined using the Monta Carlo technique with the randomisation variability test of local coefficients and the coefficient of variations of local coefficients^68–70^. In the GWR model, the location-wise coefficients of the selected wavelets are estimated with distance-decay spatial weights. The GWR model for estimating the geographically local relationships is computed as:

1 $$\begin{aligned} s=\beta _0 (\varvec{u}) + \sum _{i=1}^{m} \beta _i (\varvec{u}) w_i + \epsilon \end{aligned}$$

where *s* is the observation of a soil property (e.g. organic carbon) at the location $\varvec{u}$, $w_i(i=1,\ldots ,m)$ is the *i*th selected optimal wavelet at the location $\varvec{u}$, $\beta _i (\varvec{u})$ is the location-wise regression coefficient, and $\epsilon$ is the normally distributed random error. The spatially adaptive Gaussian kernel function is applied in the weighting scheme, where the optimal bandwidth is determined through the adaptive process, and the number of neighbour observations is optimised by minimising the AICc of the model^32^.

### Model comparison and validation

We compared the WGWR to PLSR and WLR. Our implementation of PLSR used the iterative singular value decomposition algorithm. The explanatory variables in the PLSR are the selected optimal combination of the PLS components of wavelet transformed spectra. To select the optimal number of PLS factors to use in the regression we used a cross validation and selected as many factors as necessary to produced the the smallest error^71^. For the WLR, the selection of the optimal wavelet coefficients to use was the same as that for the WGWR (see above).

The methods were evaluated with an external validation process. It involved selecting, at random, 70% of the observations to train the models and the remained 30% of the observations to test the estimates. To evaluate the performance of the methods we used the coefficient of determination (R$^2$), the mean absolute error (MAE) to assess bias and root mean squared error (RMSE) to assess inaccuracy. In the cross validation, values of soil properties have been back-transformed, since they have been transformed before modelling. To further compare AICc values of different models, relative likelihood of the models was computed as:

2 $$\begin{aligned} \eta _j = exp \left(\frac{AICc_{min} - AICc_j}{2} \right) \end{aligned}$$

where $\eta _j$ and $AICc_j$ are the relative likelihood and AICc value of *j*th model, respectively; and $AICc_{min}$ is the minimum AICc value among optional models. The $\eta _j$ is used to explain the probability that the minimised information loss in the *j*th model^72^.

All computations were performed in the R software version 4.0.3^73^. The wavelet analysis was performed using the package “waveslim”^63^, the PLSR was performed using the package “pls”^74^, and the GWR was performed using the package “spgwr”^75^.
